# FOSTERING ENGAGEMENT: AN EXPLORATION OF INNOVATIVE DEMENTIA ENVIRONMENTS FROM FOUR COUNTRIES

**DOI:** 10.1093/geroni/igae098.2041

**Published:** 2024-12-31

**Authors:** Hilde Verbeek

**Affiliations:** Chair

## Abstract

Dementia is one of the major age-related diseases and challenges those affected, societies, as well as health care systems worldwide. In an effort to better meet the needs of people living with dementia (PlwD), innovative care concepts for community-dwelling PlwD, as well as those in need for long-term care are being developed worldwide. Aiming to strengthen independence and slow down the cognitive decline, efforts concentrate on continuously engaging PlwD in activities of daily life, despite a progression of the disease. This international symposium will provide five presentations on different innovative care environments in the community, which stimulate and support an active daily life of PlwD. The first presentation dissects the care environment of Green Care Farms in the Netherlands and its effects on the engagement of residents. The second presentation discusses activity programming and self-directed behavior in Canadian long-term care. The third presentation presents social participation of PlwD in shared housing arrangements in Germany. The fourth presentation describes how the neighborhood-built environment can contribute to social health aspects for PlwD in German municipalities and the fifth presentation considers the feasibility and effectiveness of a virtual care farm activity in the United States.

